# Safeguarding digital livestock farming - a comprehensive cybersecurity roadmap for dairy and poultry industries

**DOI:** 10.3389/fdata.2025.1556157

**Published:** 2025-04-16

**Authors:** Suresh Neethirajan

**Affiliations:** ^1^Faculty of Agriculture, Agricultural Campus, Dalhousie University, Truro, NS, Canada; ^2^Faculty of Computer Science, Dalhousie University, Halifax, NS, Canada

**Keywords:** cybersecurity, digital livestock farming, big data, Internet of Things (IoT), AI-driven analytics, blockchain, biosecurity, food supply chain resilience

## Abstract

The rapid digital transformation of dairy and poultry farming through big data analytics and Internet of Things (IoT) innovations has significantly advanced precision management of feeding, animal health, and environmental conditions. However, this digitization has simultaneously escalated cybersecurity vulnerabilities, presenting serious threats to economic stability, animal welfare, and food safety. This paper provides an in-depth analysis of the evolving cyber threat landscape confronting digital livestock farming, examining ransomware incidents, hacktivist interference, and state-sponsored cyber intrusions. It critically assesses how compromised digital systems disrupt critical farm operations, including milking routines, feed formulations, and climate control, profoundly impacting animal health, productivity, and consumer trust. Responding to these challenges, we present a comprehensive cybersecurity roadmap that integrates established IT security practices with agriculture-specific requirements. The roadmap emphasizes advanced solutions, such as AI-driven anomaly detection, blockchain-based traceability, and integrated cybersecurity-biosecurity frameworks, tailored explicitly to safeguard livestock farming. Additionally, we highlight human-centric elements such as targeted workforce education, rural cybersecurity capacity building, and robust cross-sector collaboration as indispensable components of a resilient cybersecurity ecosystem. By synthesizing technical advancements, regulatory perspectives, and socio-economic insights, the paper proposes a proactive strategy to enhance data integrity, secure animal welfare, and reinforce food supply chains. Ultimately, we underscore that effective cybersecurity is not merely a technical consideration but foundational to ensuring the sustainable, ethical, and trustworthy advancement of livestock agriculture in a data-driven world.

## 1 Introduction

The 21st century has seen a shift in agriculture comparable to the machinery boom of the Industrial Revolution. Traditional dairy and poultry practices, once heavily reliant on human labor, localized knowledge, and physically intensive husbandry, now leverage sophisticated digital infrastructures to enhance productivity and oversight (Neethirajan and Kemp, 2021; Neethirajan, 2023a). Automated milking robots in dairy barns continuously gather cow health metrics, while precision feeding systems in poultry houses meticulously adjust feed rations based on granular data about flock growth and health parameters. These technologies are further supported by IoT (Internet of Things) sensors (Neethirajan, 2023b), remote health monitoring devices, climate control units, and AI-driven veterinary interventions, all of which fuel an era labeled “smart farming” or “Agriculture 4.0.” This integrated approach boosts efficiency, bolsters animal welfare, mitigates environmental impact, and fosters transparent farm-to-fork supply chains.

Despite these gains, the rapid expansion of digital platforms and real-time data transfer imposes new cybersecurity pressures (Neethirajan, 2023c). Dairy and poultry farms now function as cyber-physical systems susceptible to malicious incursion. The motivations behind these incursions can be diverse—ranging from straightforward ransomware schemes for profit to hacktivist agendas spotlighting industrial livestock concerns, and even espionage seeking proprietary genetic data. The outcomes may be catastrophic, such as sudden disruptions of milking routines that degrade animal welfare and productivity, tampered climate control systems that decimate bird populations, manipulated data that jeopardizes food quality, or strategic interference in logistics that disrupts supply chains. While other sectors (finance, healthcare, energy) have long recognized cybersecurity's importance, the role of digital threat management in contemporary animal agriculture remains relatively nascent (Latino and Menegoli, 2022; Alahmadi et al., 2022). Farmers, industry groups, regulators, and researchers increasingly face the formidable task of understanding these digital vulnerabilities, sustaining regulatory compliance, and preserving food systems' ethical and operational integrity.

This review, therefore, adopts a comprehensive lens on cybersecurity in digital livestock contexts. By centering on the dairy and poultry industries—pillars of global protein production—we identify patterns and approaches applicable to the broader agri-food sphere. The manuscript dissects the technologies underpinning modern operations, explores the diverse cybersecurity threats, evaluates the repercussions of breaches, investigates existing security measures, highlights promising new strategies, examines the synergy between cybersecurity and biosecurity, emphasizes human-centric solutions, discusses policy frameworks, underscores cross-sector collaboration, and pinpoints research gaps. Ultimately, this analysis strives to deliver pragmatic and strategic direction for stakeholders aiming to fortify agriculture's digital future.

### 1.1 Methodology

We performed a targeted literature search across prominent academic databases—Web of Science, Scopus, and Google Scholar—using key phrases such as “cybersecurity,” “digital agriculture,” “smart farming,” “livestock,” “dairy,” and “poultry.” Only English-language materials published between 2015 and 2025 were considered, ensuring currency in digital farming and cybersecurity discourse. Both empirical and conceptual works were deemed eligible if they addressed cybersecurity challenges or solutions pertaining specifically to dairy or poultry operations. Following an initial abstract screening to confirm relevance, we conducted full-text reviews to verify compliance with our inclusion criteria. Extracted information included study objectives, methodologies, primary findings, and key recommendations. Quality was appraised via established tools, such as the CASP checklist for qualitative research and PRISMA standards for systematic reviews, ensuring robust methodological insight. Data were synthesized thematically, capturing recurring topics, consensus points, and contested perspectives in the literature. This structured approach guarantees that our review consolidates the most pertinent and methodologically sound findings, offering an informed basis for the ensuing discussions.

#### 1.1.1 Background and scope

Dairy and poultry production serve as anchors in global nutrition. Dairy products (e.g., milk, cheese, yogurt) and poultry goods (e.g., eggs, chicken meat) deliver critical proteins and micronutrients worldwide. Over recent decades, forces of specialization, intensification, and worldwide market integration have reshaped these industries, prompting many farms—big and small—to adopt digital innovations aimed at boosting production, lowering costs, and meeting rising demands for welfare-friendly, high-quality outputs. However, these same digital innovations introduce novel risk factors absent from more traditional farming risk analyses.

Recognizing the significance of these technologies' vulnerability to breaches is paramount. Cyber threats, which can compromise data or disrupt automated processes, carry broader consequences for product integrity, consumer trust, and market stability. In assembling a globally oriented review, we incorporate diverse contexts, including varying regulations, economic conditions, and cultural norms, but acknowledge that the fundamental digital infrastructure now permeating dairy and poultry operations can be exploited anywhere. Through rigorous engagement with academic studies, industry reports, and policy briefs, we aim to recommend evidence-based strategies adaptable to multiple regions, ensuring a robust framework for advancing secure, resilient agricultural production.

#### 1.1.2 Digital transformation in dairy and poultry farming

The digital transformation in livestock farming constitutes a systemic overhaul of decision-making and operational routines. In dairy contexts, labor-intensive milking parlors are replaced by automated milking systems that collect continuous data on milk composition and yield (Bhoj et al., 2022). This data is then integrated with herd management software tracking feed intake, movement, rumination, and health parameters, facilitating AI-driven predictions of disease or optimal breeding intervals (Antanaitis et al., 2023). Parallelly, in the poultry domain, climate controls and precision feeding mechanisms optimize environments to yield higher feed conversion efficiencies (Moss et al., 2021). Automated egg handling minimizes manual interventions, preserving biosecurity and maintaining product quality.

Moreover, many poultry and dairy operations employ IoT sensors and machine vision systems to detect indicators of discomfort or illness. In real-time, veterinarians and nutritionists can analyze these data streams through cloud-based dashboards, making instantaneous recommendations (Gebresenbet et al., 2023). While these digital systems significantly elevate productivity and reduce errors, every connected sensor or analytics platform represents a potential entry point for attackers. This vulnerability magnifies the urgency to address cybersecurity in a manner that considers the biological nuances of livestock health and farm management.

#### 1.1.3 Defining critical infrastructure in agriculture

Digital agriculture now functions similarly to other critical infrastructure sectors—like electricity or water supply—that societies depend on for normalcy and wellbeing. Interrupting dairy or poultry pipelines could spark consumer panic, distort market prices, and sow distrust in the reliability of food products (Khan et al., 2022; Ma et al., 2021; Manning, 2023). Viewing agriculture through this lens underscores the systemic repercussions of data manipulation or system sabotage. Governments and international organizations must treat cybersecurity in farming as a collective responsibility, akin to animal disease control or environmental regulation. A single cyber incident at a major dairy or poultry operation might reverberate across supply chains, posing not just localized disruptions but cascading risks to national and global food security.

#### 1.1.4 Purpose and structure of the review

Our primary goal is twofold: first, to assess the present state of cybersecurity in digital livestock contexts, examining the degree of vulnerability in dairy and poultry farms; and second, to chart a forward-looking strategy that merges ethical, technical, and regulatory considerations to protect these essential industries from ever-evolving cyber threats. By approaching cybersecurity as an ethical and societal necessity—rather than a mere technical add-on—we emphasize the broad impacts on consumer trust, livestock welfare, and overall agrarian resilience.

This review begins by elaborating on the technological backbone enabling dairy and poultry digitalization, illuminating the sensors, AI algorithms, and data flows pivotal to current practices. We then scrutinize the cyber threat environment, dissecting potential attacker motives, common vulnerabilities, and real incidents that highlight potential damage. Subsequently, we explore the ramifications of breaches, from direct financial losses to extended harm such as compromised animal health and eroded consumer faith. We proceed to examine existing security frameworks and standards, noting both effective practices and glaring gaps. Building from these insights, we showcase advanced and emerging defenses—like AI-based anomaly detection, blockchain-trusted traceability, and integrated biosecurity-cybersecurity protocols—that can reinforce a farm's resilience. Recognizing human elements as pivotal, we delve into workforce education, rural capacity building, and cross-sector engagement. Finally, we address policy landscapes, standardization, and future research imperatives, culminating with actionable recommendations to foster a secure digital ecosystem in livestock farming. Our intent is to guide farmers, industry stakeholders, policymakers, and researchers in uniting around comprehensive, context-aware cybersecurity solutions—thereby ensuring that Agriculture 4.0 remains sustainable, responsible, and equitable.

## 2 The technological landscape of digital livestock farming

The intricate suite of digital tools now embedded in dairy and poultry operations has reshaped the very essence of farm management. Recognizing these technologies is crucial for understanding the genesis of cyber threats and formulating effective safeguards.

### 2.1 Key technologies in dairy production

#### 2.1.1 Automated milking systems (AMS)

Automated Milking Systems alleviate labor constraints and rigid milking schedules by allowing cows to enter milking stations on their own. Robotic arms oversee teat sanitation, attachment, and milk extraction, relaying data on yield, conductivity (a mastitis indicator), and milking intervals to herd management software. Yet AMS hinge on precise sensors and uninterrupted connectivity. A cyberattack (Anton et al., 2024; Vatn et al., 2023) tampering with conductivity readings could conceal infections, undermining herd health and eventual productivity. Likewise, disabling AMS functionality could create discomfort, disrupt lactation, and erode revenue.

#### 2.1.2 Herd management software and sensors

Herd management platforms centralize diverse metrics, from feed consumption and body condition scores to locomotion data and rumination patterns. This consolidated information supports refined feeding plans, estrus detection, and health interventions. However, a compromised sensor may feed false data into the system, risking overmedication or missed signs of genuine illness. Securing data authenticity, confidentiality, and reliable access is non-negotiable when a single corrupted data stream could derail strategic decisions.

#### 2.1.3 Environmental control systems

Dairy barns increasingly depend on automated fans, sprinklers, heating, and lighting to preserve cow wellbeing and productivity. Penetrating these systems may prompt heat stress on scorching days or inflict cold stress under frigid conditions. Disruptions to environmental controls can quickly escalate from productivity setbacks to serious welfare concerns, amplifying both ethical and operational risks.

### 2.2 Key technologies in poultry farming

#### 2.2.1 Automated feeding and egg collection systems

In poultry houses, automated feeders supply customized rations for peak nutrient efficiency, while egg conveyors cut down on contamination and labor. Attacking these systems might corrupt feed formulas—potentially introducing toxins—or delay egg retrieval, resulting in spoilage and breakage. Such scenarios threaten just-in-time supply practices and consumer expectations of product availability.

#### 2.2.2 Climate control and lighting automation

Poultry welfare hinges on controlled lighting schedules and stable indoor climates. Automation adjusts temperature, ventilation, and humidity, with lighting cycles that dictate laying patterns. An assailant could, for instance, over-activate fans to chill flocks or fail to ventilate during extreme heat, causing devastating losses. Tampering with lighting sequences may trigger erratic bird behavior and lower egg output, illustrating how digital reliability links directly to biological outcomes.

#### 2.2.3 Precision livestock farming tools for bird health monitoring

Vision systems, microphones, and wearables are often combined with AI to detect signs of disease or discomfort. By flagging potential health issues early, these tools reduce antibiotic use, protect public health, and bolster animal welfare. However, cyberattacks might silence legitimate alarms, inject spurious alerts that waste resources, or obscure genuine pathogen outbreaks. Safeguarding these monitoring systems is fundamental to realizing precision farming's promises.

### 2.3 IoT and cloud platforms in agriculture

**Figure 1:**
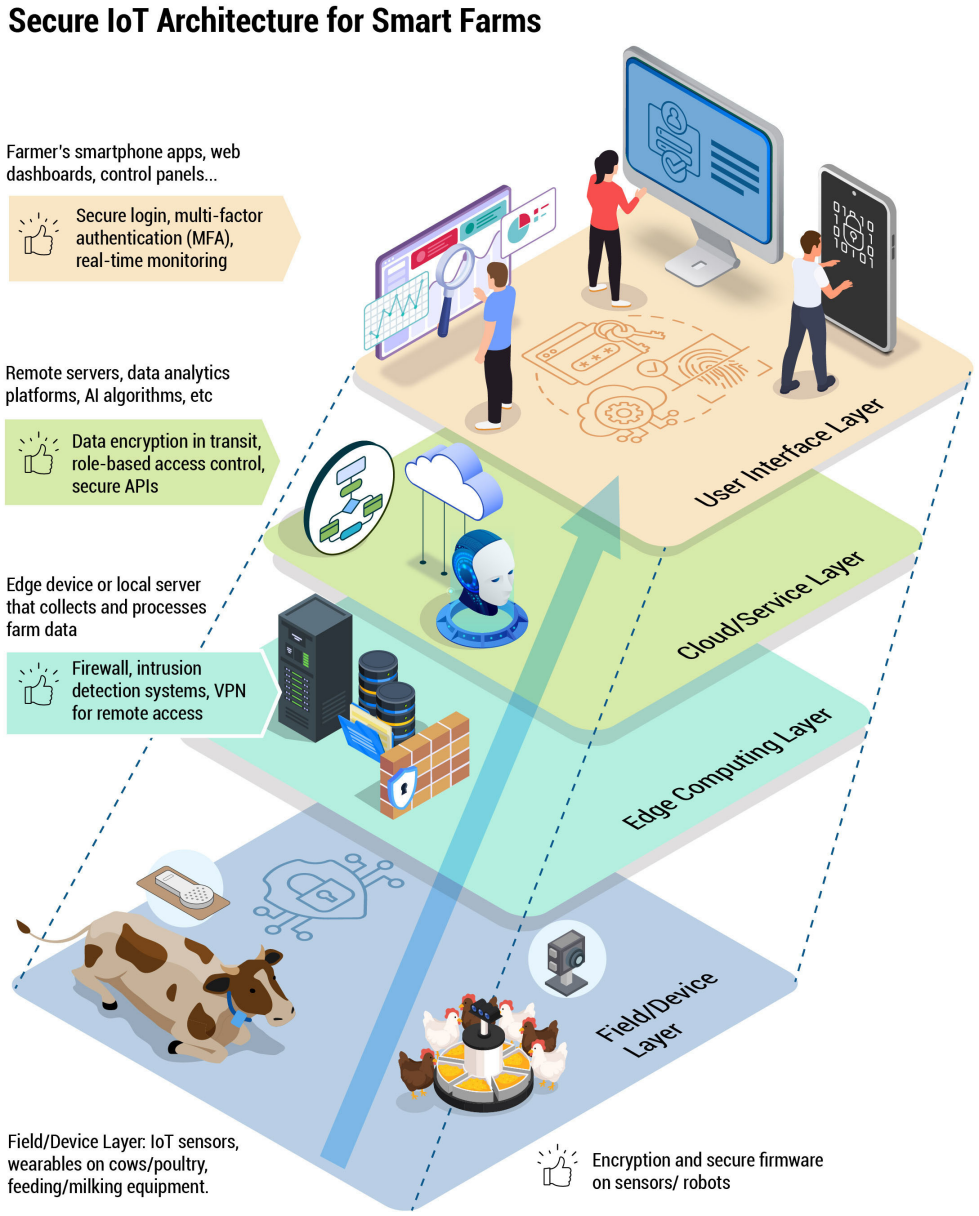
A multi-layer IoT architecture model illustrating how data moves from the field/device layer (sensors, wearables, feeding/milking equipment) through edge computing nodes and cloud services, finally reaching user interfaces. Each layer includes recommended security features—such as encryption, secure firmware, firewalls, and intrusion detection systems—designed to protect the integrity of dairy and poultry operations.

Across agriculture, IoT devices appear in feed bins, milking tanks, and drones, all funneling data to cloud-based services for troubleshooting, big data analytics, and predictive modeling. Each endpoint, if not rigorously protected, can become a launchpad for infiltration. From encrypted communications and secure firmware patches to robust device identity checks, multiple strategies are needed to keep networks resilient. Moreover, cloud providers must uphold strict security protocols because a single breach could cascade across many interconnected farms. Figure 1 depicts a secure IoT blueprint for livestock operations, outlining key security measures at each layer.

Figure 1 presents an original IoT architecture model specifically designed for digital livestock farming. It comprises four interconnected layers: the Field/Device Layer, Edge Computing Layer, Cloud/Service Layer, and User Interface Layer. Each layer addresses unique operational needs and faces distinct cybersecurity threats, requiring tailored protective measures. The Field/Device Layer collects data on animal health, environmental conditions, and operational metrics, making it vulnerable to physical tampering, firmware vulnerabilities, and data manipulation. Recommended countermeasures include tamper-resistant hardware, secure firmware updates, and robust encryption protocols. Unlike generic IoT models, this framework emphasizes sector-specific considerations such as animal welfare, real-time monitoring, and operational continuity.

The subsequent layers further support data processing and decision-making. The Edge Computing Layer processes data locally to minimize latency for real-time decisions, but faces risks such as unauthorized access, data interception, and malware. Countermeasures here involve secure gateways and local intrusion detection systems. The Cloud/Service Layer facilitates large-scale data storage and analytics, exposing it to cloud misconfigurations, data breaches, and Denial-of-Service (DoS) attacks. End-to-end encryption, role-based access control (RBAC), and routine security audits are essential protections. Lastly, the User Interface Layer allows operators to manage the farm remotely, presenting risks from social engineering and weak authentication practices. Multi-factor authentication (MFA) and cybersecurity awareness training provide effective defense strategies at this level.

### 2.4 Data-driven decision making and predictive analytics

Reliance on data analytics allows farmers to foresee disease outbreaks, fine-tune breeding timing, and forecast market demand. Leveraging AI models, they interpret both historical and live data to steer decision-making. However, any corruption of this data—be it the insertion of false inputs or tampering with existing records—undermines model accuracy. Tainted datasets could lead to detrimental culling decisions, flawed feeding recommendations, or genetic setbacks. Guaranteeing data integrity is thus pivotal for maintaining confidence in AI-based agriculture.

### 2.5 Integration with broader agri-food supply chains

Dairy and poultry goods traverse a complex web of processors, distributors, retail outlets, and end consumers. Digital traceability tools enable farm-to-fork transparency, building consumer trust around origin and authenticity. Yet, interlinked supply chains also raise the stakes: a single hacked logistics node or compromised data broker can produce counterfeit records, trigger product recalls, or sow market panic. Collaboration is therefore paramount—securing farm systems alone is insufficient without a synchronized, sector-wide cybersecurity strategy.

## 3 The cyber threat landscape in dairy and poultry operations

### 3.1 Ransomware attacks

Ransomware attacks involve malicious actors encrypting critical farm data or locking essential digital systems, rendering them inaccessible until a ransom is paid. Attackers typically exploit vulnerabilities through phishing emails or compromised software updates, causing severe operational disruptions such as halted milking processes or interrupted feed distribution schedules. Farms are particularly vulnerable due to their reliance on real-time data and automated systems, making rapid recovery essential to avoid animal welfare crises and economic losses.

### 3.2 Hacktivism

Hacktivist attacks are motivated primarily by ideological or political objectives rather than financial gain. Activists target digital livestock operations to protest perceived ethical issues, such as animal welfare concerns or environmental impacts of intensive agriculture. Common tactics include Distributed Denial-of-Service (DDoS) attacks, website defacements, unauthorized data disclosures, and leaking sensitive farm operation footage. Such incidents can severely damage reputation, consumer trust, and regulatory compliance.

### 3.3 State-sponsored espionage and sabotage

State-sponsored cyberattacks involve sophisticated actors supported by national governments aiming to disrupt critical infrastructure or obtain strategic advantages through espionage. In agriculture, these attacks may target proprietary genetic data repositories, precision farming algorithms, or critical control systems managing feed formulation and environmental conditions. Such strategic intrusions can cause widespread supply chain disruptions or economic instability during geopolitical tensions, highlighting agriculture's status as critical infrastructure vulnerable to hybrid warfare tactics.

### 3.4 Motivations behind attacks

#### 3.4.1 Financially motivated ransomware attacks

Ransomware groups seek easy payoffs (Connolly and Borrion, 2022; McIntosh et al., 2021). Targeting a high-throughput dairy or poultry operation, they know that hours of downtime yield irreversible losses—milk not harvested on time, birds overcrowded or starving. These conditions force quick ransom payments. The success of past attacks in other sectors shows criminals that agriculture, with limited cybersecurity maturity (Kulkarni et al., 2024; Rijswijk et al., 2021), can be fertile ground for extortion.

#### 3.4.2 Hacktivist and activist threats targeting animal welfare issues

**Figure 2:**
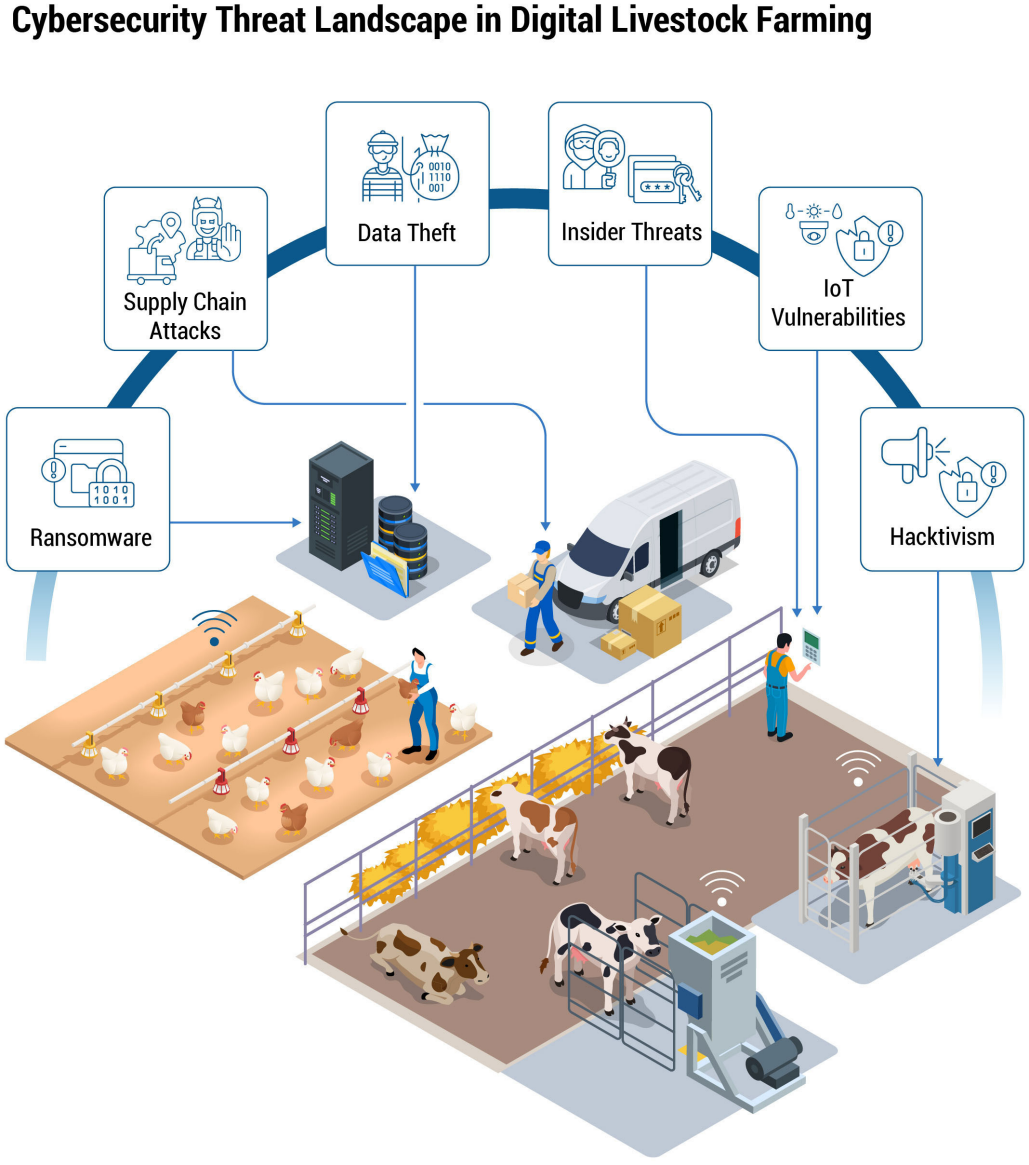
A conceptual overview of the main cyber threats in digital livestock operations, highlighting ransomware attacks, data theft, insider threats, IoT vulnerabilities, hacktivism, and supply chain attacks. The illustration shows how these threats can target poultry and dairy environments, from automated feeding lines to milking robots, as well as interconnected logistics and data storage systems.

Some attackers may hold strong ideological stances (Melnyk et al., 2022; Shinde, 2021) against industrial livestock production. By manipulating environmental controls or releasing sensitive video footage from inside barns, they aim to publicly shame the industry, prompt regulatory action, or sway consumer behavior. While not financially motivated, such attacks can be deeply disruptive and challenge farmers to justify their practices and invest in greater transparency. As illustrated in Figure 2, digital livestock operations face a broad range of cyber threats, from ransomware to hacktivism, which can disrupt both supply chains and on-farm systems.

#### 3.4.3 State-sponsored threats and critical infrastructure disruption

In a world of geopolitical rivalries, food systems can be leveraged as strategic assets (Metta et al., 2022; Brassesco et al., 2022; Syed et al., 2022). A hostile state might quietly infiltrate systems controlling feed supplies, waiting to sabotage them during diplomatic crises, causing shortages or quality issues that erode public faith in governance. Though speculative, the increasing digitization of agriculture makes such scenarios more plausible, prompting national security circles to consider agricultural cybersecurity as part of critical infrastructure protection. Table 1 provides a detailed cybersecurity risk assessment matrix tailored specifically for dairy and poultry farming, highlighting potential threats alongside their likelihood, severity, and suggested mitigation measures. While threats such as ransomware, phishing, and Denial-of-Service (DoS) attacks are widely recognized, certain attack types warrant additional contextualization. For instance, “Supply chain attacks” involve compromising third-party vendors or suppliers, indirectly enabling unauthorized system access. “AI/ML manipulation” refers to adversarial interference with artificial intelligence or machine learning-based decision systems, potentially resulting in detrimental operational outcomes. “Remote access exploitation” encompasses unauthorized intrusion into remote connectivity channels, allowing attackers to gain direct control over farm operations. “Cloud security breaches” describe incidents involving compromised cloud platforms, exposing critical farm data to external threats. Lastly, “Insider threats” represent vulnerabilities arising from intentional or accidental actions of internal personnel, including sabotage or unintended data disclosures. Understanding the specific nature of these cyber threats underscores their potential implications for precision agriculture operations and supports the formulation of effective cybersecurity measures.

**Table 1:** Risk assessment matrix for dairy and poultry industry cybersecurity.

**Threat type**	**Likelihood**	**Impact**	**Mitigation strategies**
Ransomware attacks	High	High - Can halt operations, cause financial losses, and potentially lead to animal deaths	Implement regular backups, train staff on cybersecurity, use multi-factor authentication
IoT device vulnerabilities	High	High - Can compromise critical systems controlling feeding, milking, and environmental conditions	Implement secure-by-design IoT frameworks, regular firmware updates, network segmentation
Supply chain attacks	Medium	High - Can affect multiple farms through compromised vendor software	Vet cybersecurity credentials of vendors, implement secure data-sharing agreements, conduct regular audits
Data theft and espionage	High	Medium - Can lead to loss of competitive advantage and privacy breaches	Encrypt sensitive data, implement strong access controls, monitor data access patterns
AI/ML manipulation	Medium	High - Can lead to incorrect decisions affecting animal health and productivity	Ensure model explainability, robust validation of training data, continuous monitoring of ML outputs
Insider threats	Medium	High - Can cause intentional damage to systems or data leaks	Implement strict access controls, conduct regular security awareness training, monitor user activities
Denial-of-Service (DoS) attacks	Medium	High - Can disrupt critical farm operations and animal care	Implement network segmentation, use DoS protection services, have backup communication channels
Phishing attacks	High	Medium - Can lead to unauthorized access and data breaches	Conduct regular phishing awareness training, implement email filtering solutions, use secure authentication methods
Unpatched software vulnerabilities	High	High - Can allow attackers to exploit known weaknesses	Implement regular patching schedules, use virtual patching for legacy systems, conduct vulnerability assessments
Remote access exploitation	Medium	High - Can lead to unauthorized control of farm systems	Implement VPNs, multi-factor authentication, and strict access policies for remote connections
Cloud security breaches	Medium	High - Can compromise large amounts of centralized farm data	Use encryption for data in transit and at rest, implement strong access controls, regularly audit cloud security
Social engineering attacks	High	Medium - Can lead to inadvertent disclosure of sensitive information	Conduct regular security awareness training, implement strict information sharing policies
Physical security breaches	Low	High - Can lead to direct tampering with farm equipment and systems	Implement physical access controls, surveillance systems, and integrate with cybersecurity measures
Compromised traceability systems	Medium	High - Can undermine food safety and supply chain integrity	Implement blockchain-based traceability, regular audits of data integrity, redundant record-keeping
Bioterrorism-linked cyber attacks	Low	Extreme - Can cause widespread harm to animal and human health	Integrate cybersecurity with biosecurity measures, implement advanced threat detection systems

### 3.5 Attack vectors and vulnerabilities

#### 3.5.1 IoT device exploits

Many IoT devices were never designed with stringent security in mind. Default admin passwords, unencrypted data channels, or the inability to patch firmware promptly (Chantzis et al., 2021) enable attackers to commandeer devices easily. Farmers must demand security-by-design from vendors and adopt stringent procurement criteria.

#### 3.5.2 Weak authentication and password management

It only takes one weak credential to open the door to a network. Simple passwords shared among employees, failure to rotate credentials, or overlooking two-factor authentication leave valuable systems exposed (Jariwala, 2023). Better authentication schemes and education can drastically reduce these low-effort attacks.

#### 3.5.3 Legacy systems and unpatched software

Legacy control units managing feeding lines or milking machinery may run outdated operating systems with known exploits. Replacement or retrofitting may be costly, but ignoring these vulnerabilities is risky. Regular patching, virtual patching (through network segmentation and intrusion prevention systems), and planning gradual equipment upgrades are necessary steps.

#### 3.5.4 Remote access and third-party technology management risks

Cloud services, remote maintenance tools, and data brokers streamline operations but widen the attack surface (Darwish, 2024; Mattsson, 2023). Compromising a trusted service provider can yield control over multiple client farms. Vendor contracts should include security clauses, audits, and contingency plans. Trust must be earned, monitored, and revocable if standards aren't met.

Figure 2 provides a conceptual overview of major cybersecurity threats targeting digital livestock operations, categorizing them into ransomware attacks, hacktivism, and IoT vulnerabilities, each contextualized specifically for dairy and poultry farms. Ransomware attacks present a direct threat by encrypting critical farm data, potentially halting essential processes like milking and feed distribution until a ransom is paid. Hacktivism involves ideologically driven individuals or groups who may disrupt operations by tampering with environmental controls or leaking sensitive farm footage to damage public perception and trust.

Additionally, IoT vulnerabilities represent a significant risk, as unsecured or poorly configured devices could be exploited as entry points, allowing malicious actors to manipulate operational data or compromise system controls. Such vulnerabilities can severely affect animal health, operational efficiency, and food safety. Understanding these threats within the context of dairy and poultry environments highlights the necessity of targeted cybersecurity strategies, including secure IoT design, rigorous data protection measures, and comprehensive incident response planning.

### 3.6 Case studies of cyber incidents in agriculture

The agricultural sector has become an enticing target for cybercriminals, with recent high-profile attacks exposing the vulnerability of our food supply chain. In 2021, a devastating ransomware attack paralyzed JBS Foods, the world's largest meat processor, forcing a complete shutdown of US beef plants and disrupting poultry and pork production. This incident, which resulted in an $11 million ransom payment, sent shockwaves through the industry and highlighted the critical need for robust cybersecurity measures. But it's not just meat producers at risk. The same year, the BlackMatter ransomware group strategically targeted New Cooperative, an Iowa-based grain company, during the crucial harvest season (Hartley, 2022; Hazrati et al., 2022). This attack threatened to cripple grain storage and animal feed operations, demonstrating how cybercriminals can exploit agricultural cycles for maximum impact. Even the dairy industry isn't immune, as evidenced by a cyber incident at Schreiber Foods that led to a widespread cream cheese shortage (Cinar and Thomas, 2023), proving that localized attacks can have far-reaching consequences for consumers. These incidents underscore the diverse and evolving nature of cyber threats in agriculture. From meat processing to grain storage, and from equipment manufacturing to dairy production, no segment of the industry is safe. As attackers become more sophisticated and opportunistic, the potential for significant operational, financial, and supply chain disruptions grows. The agricultural sector must recognize that cybersecurity is no longer optional – it's a critical component of ensuring food security and maintaining consumer trust in an increasingly digital world.

### 3.7 Regulatory and reporting challenges

Without mandatory reporting frameworks, many farms quietly pay ransoms or rebuild systems without public disclosure (Logue and Shniderman, 2021; Balaji et al., 2023). This secrecy impedes collective learning and prevents a comprehensive threat picture from emerging. Policymakers must consider incident reporting mandates, safe harbor protections for those reporting incidents, and trusted clearinghouses for cybersecurity intelligence. Such measures encourage transparency, facilitate benchmarking, and drive continuous improvement.

#### 3.7.1 Security challenges and countermeasures in digital livestock farming

Digital livestock farming faces critical cybersecurity challenges related to data privacy, authenticity and integrity, availability, and trust management—similar to those observed in secure edge computing scenarios for smart city applications (Ajao and Apeh, 2023). These challenges highlight the urgency of developing specialized security frameworks tailored specifically for dairy and poultry environments.

Data Privacy issues arise due to sensitive information such as animal health records, production metrics, operational schedules, and proprietary business data being vulnerable to unauthorized access or leaks. Such breaches can result in regulatory non-compliance, financial loss, and compromised competitive advantage. Effective countermeasures include robust encryption methods for data both at rest and in transit, anonymization techniques prior to third-party data sharing, and strict data governance policies.

Security threats to authenticity and integrity involve unauthorized data manipulation, potentially leading to falsified sensor readings or altered operational data. This could significantly disrupt animal welfare monitoring, feeding management, or productivity outcomes. Recommended countermeasures include multi-factor authentication (MFA), digital certificates for device identity verification, secure firmware updates, and employing AI-driven anomaly detection systems for continuous monitoring.

Challenges associated with availability threaten the continuous operation of critical IoT infrastructure in dairy and poultry farms. Attacks such as ransomware or distributed denial-of-service (DDoS) could disrupt automated milking and feeding systems, environmental control mechanisms, and essential operational communications. Effective safeguards include deploying redundant backup systems, performing timely software patches, and adopting advanced intrusion detection and prevention systems alongside detailed incident response plans.

Lastly, issues surrounding trust management and policy enforcement arise from the complex relationships among farmers, agricultural technology providers (ATPs), and service providers. Mismanagement or unauthorized disclosures of sensitive farm data may erode stakeholder trust and breach privacy regulations. Countering these issues necessitates explicit consent mechanisms for data sharing, comprehensive role-based access control (RBAC), clear privacy policies, and regular cybersecurity awareness training.

Drawing insights from recent advances in secure edge computing, our framework integrates advanced cybersecurity modeling techniques, including Petri Net-based simulations and Genetic Algorithm-based Reinforcement Learning (GARL), to optimize anomaly detection, enhance system resilience, and effectively mitigate threats (Ajao and Apeh, 2023). These methodologies enable proactive threat management, ensure secure authentication and authorization, and enhance system resilience against cybersecurity breaches, thereby ensuring the sustainability and operational continuity of digital livestock systems.

## 4 Impact of cybersecurity incidents on dairy and poultry industries

### 4.1 Operational disruptions and economic losses

Milking cannot be “postponed” indefinitely; feed must be delivered on schedule; eggs cannot remain uncollected without spoilage risk. Cyber incidents disrupt these finely tuned production rhythms. Lost production equates to lost income, but downtime also engenders downstream costs: compensating staff for extra hours, disposing of spoiled inputs, hiring emergency technical support, and perhaps paying ransoms. Over time, repeated incidents could push marginal operations out of business, reducing industry diversity and resilience.

### 4.2 Food safety and quality risks

When sensor data or quality control logs are compromised, pathogens can slip through undetected. Tainted milk or eggs can reach consumers, risking outbreaks of foodborne illness. Beyond direct health impacts, the reputational blow to brands and supply chains can persist for years. Regulatory penalties, legal liabilities, and class-action lawsuits add to the financial and credibility burdens. Safeguarding data authenticity thus becomes integral to maintaining high food safety standards.

### 4.3 Animal welfare and ethical considerations

Dairy cows and poultry flocks rely on consistent care. Cyberattacks that disrupt feeding or climate controls subject animals to hunger, heat stress, respiratory distress, or disease vulnerability. Animal welfare standards, increasingly enshrined in regulations and demanded by consumers, are at risk. Farms must ensure that no single point of digital failure can cause widespread suffering. This may involve manual overrides, fail-safe modes, and dedicated backup systems that maintain minimum welfare conditions.

### 4.4 Reputational damage and loss of consumer trust

Food brands operate in an environment of intense public scrutiny. A single widely publicized cyber incident causing significant harm can erode trust not only in a brand but in the broader production methods it represents. Dairy and poultry industries must recognize that safeguarding cybersecurity is part of their social license to operate (Creese et al., 2021; Wang et al., 2021; Pollini et al., 2022). Restoring consumer confidence after a breach may require transparent communication, demonstrable reforms, and independent audits to reassure stakeholders.

### 4.5 Cascading effects on the broader supply chain

Agriculture does not operate in isolation. Feed suppliers, veterinary service providers, processors, distributors, and retailers form a tightly coupled network. If a farm's compromised data leads to feed formulation errors at the mill, that error propagates to multiple customers. If distribution schedules are altered, retailers face gaps in availability, leading to lost sales and dissatisfied consumers. Cascading failures highlight the necessity of supply chain coordination (Li and Xu, 2021; Syed et al., 2022; Squillace and Cappella, 2024; Alqudhaibi et al., 2024), information sharing, and sector-wide resilience measures.

## 5 Existing cybersecurity measures and standards

### 5.1 Current best practices in agricultural cybersecurity

Many recommended practices originate from general IT security guidelines: firewalls, antivirus software, intrusion detection, data backups, and secured Wi-Fi (Aslan et al., 2023; Jimmy, 2024). Some farms impose basic password policies or keep critical systems off the public internet. Although these steps deter low-level attacks, they may not suffice against sophisticated adversaries who can exploit specific agricultural workflows or zero-day vulnerabilities.

### 5.2 Guidelines and frameworks from government and industry bodies

While governments and industry associations have begun acknowledging the cybersecurity challenge, much guidance remains broad. The U.S. Department of Homeland Security and others have published advisories, but these rarely delve into agriculture's unique operational constraints (Hiller et al., 2024; Drape et al., 2021; Alahe et al., 2024; Yazdinejad et al., 2021). Some producer groups have issued data privacy and security principles, and the EU's GDPR protects personal data but not necessarily farm operational data or animal welfare metrics. The lack of specialized agricultural cybersecurity standards leaves farmers uncertain about which measures to prioritize (Riaz et al., 2022; Ferreira et al., 2025).

### 5.3 Cyber hygiene and basic security controls

**Figure 3:**
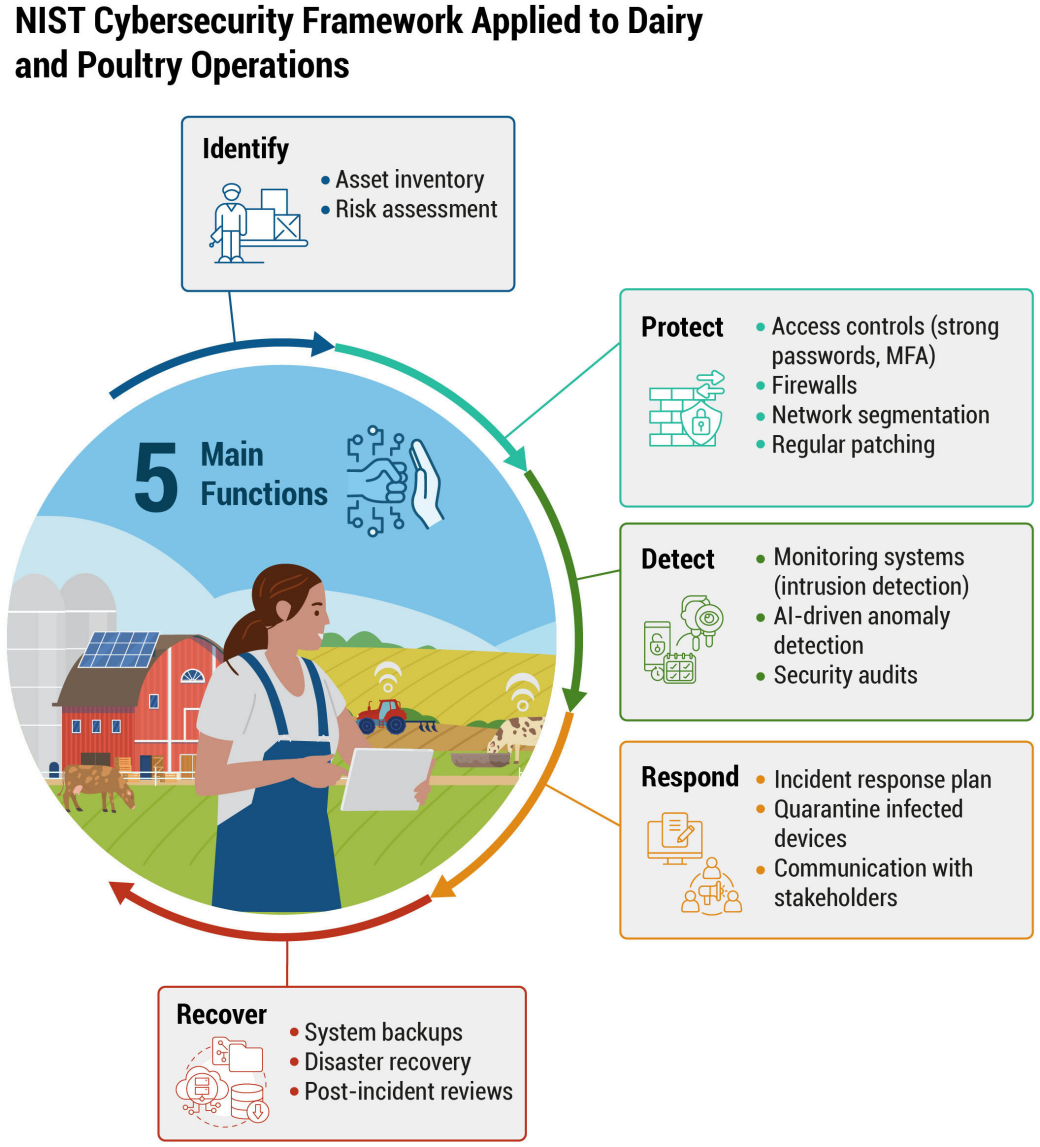
Adapting the five core functions of the NIST Cybersecurity Framework (Identify, Protect, Detect, Respond, Recover) to address specific considerations in dairy and poultry farming. Examples include asset inventory, access controls, AI-driven anomaly detection, incident response, and backup strategies—each stage tailored to operational and animal welfare needs.

Educating staff about phishing, requiring strong passwords, promptly applying vendor patches, and securing remote access with VPNs or MFA (Multi-Factor Authentication) are foundational steps. Although these actions are low-cost and high-impact, their implementation remains inconsistent, partly due to limited IT expertise in rural settings and the perception that “it won't happen here.” Initiatives like the NIST Cybersecurity Framework, adapted for the agricultural context, can serve as a foundation for aligning cybersecurity efforts across the sector. To contextualize these functions for livestock operations, we adapt the NIST Cybersecurity Framework, as shown in Figure 3.

Figure 3 adapts the five core functions of the NIST Cybersecurity Framework—Identify, Protect, Detect, Respond, and Recover—to specifically address dairy and poultry farming environments. The Identify function emphasizes creating comprehensive asset inventories of farm devices and sensors. Protect includes implementing robust security measures, such as encryption, multi-factor authentication (MFA), and strict access controls, to secure sensitive data from unauthorized access.

The Detect function highlights using AI-driven anomaly detection systems to quickly identify irregularities in operational metrics, such as unusual feed consumption patterns or unexpected changes in milk yield. Under Respond, farms should prepare tailored incident response plans focused on maintaining operational continuity and animal welfare during cyber incidents. Finally, Recover stresses the importance of establishing reliable backup and restoration processes, ensuring critical functions—such as climate control and feeding systems—can quickly resume normal operation after disruptions.

### 5.4 Limitations of existing security measures in farm environments

Generic solutions designed for offices or data centers may not translate seamlessly to farm contexts. For example, patching a milking robot's OS may require shutting down production at peak times—an unacceptable trade-off. Internet connectivity in rural areas might be too slow for frequent updates. Vendors may not provide long-term firmware support for farm-specific IoT devices. These constraints demand innovative, context-aware approaches rather than one-size-fits-all security protocols. As shown in Table 2, agriculture shares cybersecurity challenges with sectors like healthcare and aerospace, indicating that cross-industry strategies can inform more robust security measures for dairy and poultry operations.

**Table 2:** Analysis of healthcare and aerospace cybersecurity challenges and solutions for their applicability for dairy and poultry farms.

**Sector**	**Key challenges**	**Innovative solutions**	**Applicability to agriculture**	**Unique considerations**
Healthcare	Protection of sensitive patient data	Blockchain for secure health records	Securing animal health records and genetic data	Privacy regulations like HIPAA
Healthcare	Medical device vulnerabilities	AI-powered anomaly detection	Monitoring IoT devices in smart barns	FDA approval for security patches
Healthcare	Compliance with strict regulations (e.g., HIPAA)	Automated compliance monitoring tools	Adapting for food safety and animal welfare regulations	Balancing accessibility with security
Aerospace	Real-time threat detection for in-flight systems	Quantum-resistant cryptography	Securing real-time data from farm sensors and drones	High stakes of system failures
Aerospace	Supply chain integrity	Blockchain-based supply chain tracking	Ensuring traceability in food supply chains	Complex international supply networks
Aerospace	Secure communication with ground control	Advanced encryption protocols	Securing remote access to farm management systems	Vast distances and varied environments
Agriculture	Legacy system integration	Edge computing solutions	Bridging old and new farm technologies securely	Wide range of equipment ages and types
Agriculture	Limited cybersecurity awareness among farmers	Tailored training programs	Improving cyber hygiene in rural communities	Varied technical expertise levels
Agriculture	IoT device vulnerabilities in smart farming	Secure-by-design IoT frameworks	Protecting connected devices in precision agriculture	Harsh environmental conditions
Agriculture	Data privacy in precision farming	Privacy-preserving machine learning	Analyzing farm data without compromising privacy	Balancing data sharing and protection
Healthcare	Telemedicine security	Multi-factor authentication	Securing remote veterinary services	Patient confidentiality concerns
Aerospace	Autonomous system security	AI-driven intrusion detection	Protecting autonomous farm equipment	Safety-critical operations
Healthcare	Insider threats	Behavioral analytics	Monitoring access to sensitive farm data	High staff turnover rates
Aerospace	Resilience against state-sponsored attacks	Cyber deception technologies	Protecting critical agricultural infrastructure	National security implications
Agriculture	Weather-related cybersecurity risks	Climate-adaptive security protocols	Ensuring system integrity during extreme weather	Unpredictable environmental factors
Healthcare	Interoperability of secure systems	Standardized security APIs	Integrating diverse farm management systems securely	Varied vendor ecosystems
Aerospace	Secure software updates for critical systems	Over-the-air update security	Safe updates for smart farming equipment	Remote and distributed systems
Agriculture	Biosecurity and cybersecurity integration	Holistic bio-cyber risk frameworks	Unified approach to biological and digital threats	Interdependence of physical and digital security
Healthcare	Patient data anonymization	Advanced data masking techniques	Protecting farmer and livestock privacy in research	Balancing research needs with privacy
Aerospace	Predictive maintenance security	Secure IoT sensor networks	Safe monitoring of farm equipment health	Continuous operation requirements
Agriculture	Secure precision livestock farming	AI-powered behavioral monitoring	Early detection of animal health and welfare issues	Ethical considerations in animal monitoring
Healthcare	Secure health information exchange	Federated learning systems	Collaborative farm data analysis without data sharing	Competitive concerns in data sharing
Aerospace	Quantum computing threats	Post-quantum cryptography	Future-proofing agricultural data protection	Long-term data sensitivity
Agriculture	Cybersecurity for vertical farming	Integrated physical-cyber security	Protecting controlled environment agriculture	Urban setting vulnerabilities
Agriculture	Drone security in precision agriculture	Secure drone communication protocols	Safe operation of agricultural drones	Regulatory compliance for airspace use

### 5.5 Practical considerations and barriers for cybersecurity implementation in livestock farming

Despite the availability of effective cybersecurity strategies, several practical barriers complicate their implementation in dairy and poultry farming environments. One significant challenge involves limited technical expertise and specialized cybersecurity knowledge among farm operators and rural agricultural workers. Farms, particularly small- to medium-sized operations, often lack dedicated IT personnel and must rely on general farm staff or outsourced service providers who may have limited cybersecurity skills, potentially leaving vulnerabilities unaddressed.

Economic constraints present additional barriers. Advanced cybersecurity solutions, such as blockchain technologies, AI-based intrusion detection, or comprehensive data encryption, may entail significant initial costs and ongoing maintenance expenses. Smaller operations with restricted budgets may prioritize immediate production needs over perceived long-term cybersecurity benefits, reducing their willingness to invest proactively.

Infrastructure limitations, particularly inadequate or unstable internet connectivity in rural areas, also impact effective deployment of cybersecurity measures. Real-time anomaly detection systems, secure cloud backups, or timely software patching depend heavily on reliable connectivity, which can be inconsistent in remote or rural locations, thereby limiting their effectiveness or even discouraging adoption altogether.

Behavioral and cultural factors also play a crucial role. Farm operators and staff may lack adequate cybersecurity awareness or training, leading to vulnerabilities such as poor password hygiene, susceptibility to phishing attacks, or inadvertent misuse of critical systems. Resistance to change—driven by perceptions that cyber threats are unlikely or irrelevant to rural operations—further exacerbates implementation challenges.

Overcoming these barriers requires context-sensitive solutions. Stakeholders should prioritize cybersecurity education tailored specifically to agricultural workers, develop cost-effective and user-friendly technologies, and advocate for policy incentives such as subsidies, tax benefits, or favorable insurance terms for compliant farms. Collaborative approaches involving government support, industry partnerships, and targeted rural outreach initiatives can significantly enhance the adoption and sustainability of cybersecurity practices in digital livestock farming.

## 6 Advanced cybersecurity strategies and emerging technologies

### 6.1 AI-driven intrusion detection systems (IDS) and anomaly detection

**Figure 4:**
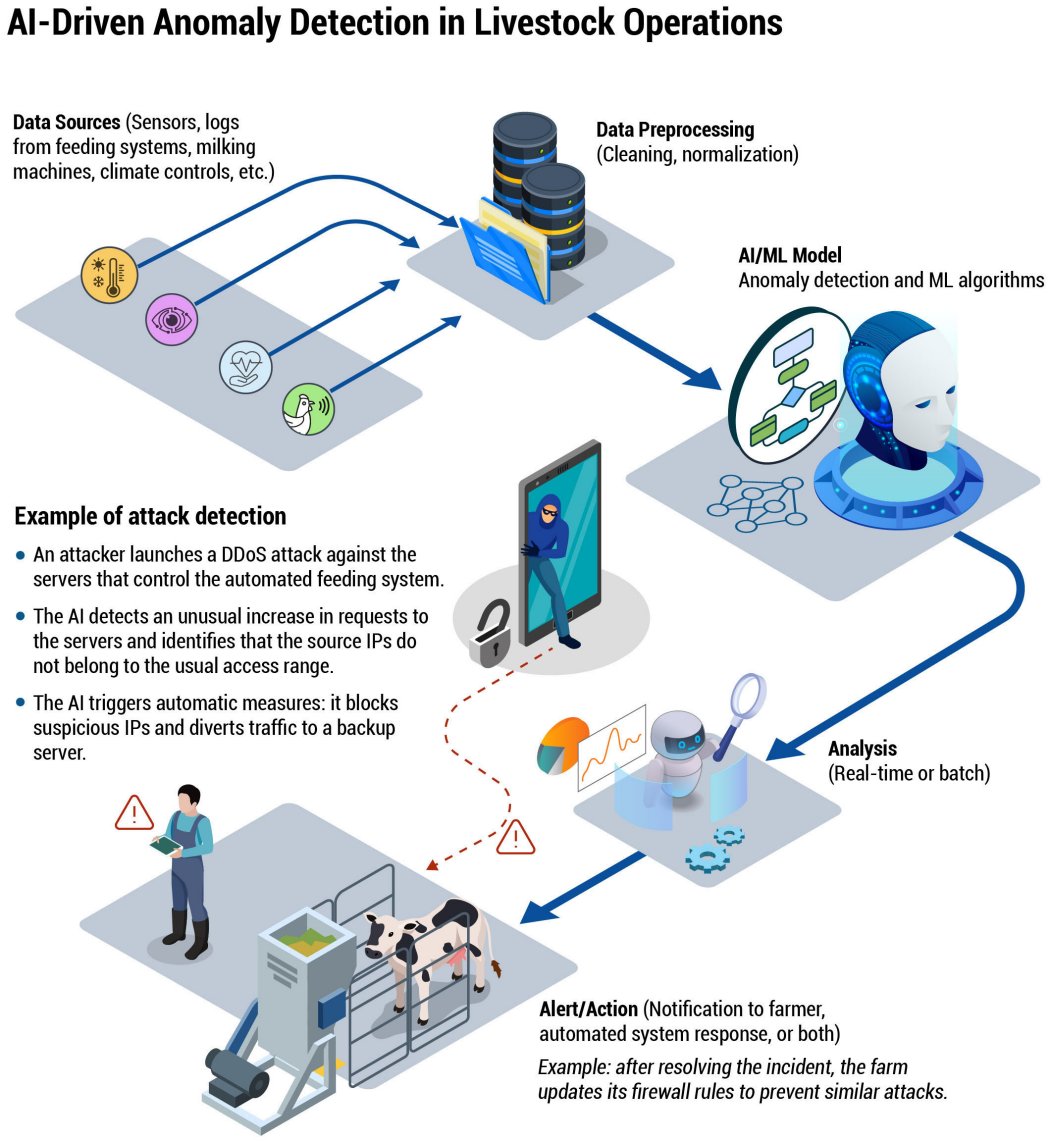
A flowchart demonstrating how an AI/ML system ingests data from various farm sources (e.g., feeding logs, milking robots, climate controls), preprocesses it, and then applies anomaly detection algorithms. In the event of a detected threat—such as a DDoS attack on feeding systems—the AI can automatically block malicious IPs and alert farm operators, emphasizing real-time or batch analysis for proactive defense.

Machine learning can establish behavioral baselines for milking cycles, feeding patterns, and environmental parameter fluctuations. Deviations from norms—such as unusual temperature spikes at midnight or abrupt drops in milk yield data—can alert administrators to possible intrusions. However, these ML models must be robust against adversarial examples and data poisoning attempts. Continuous model validation, retraining, and the incorporation of domain knowledge from veterinarians and animal scientists can enhance IDS effectiveness. As seen in Figure 4, AI-driven anomaly detection processes incoming sensor and log data, allowing the system to identify and respond to threats such as DDoS attacks in real time.

### 6.2 Blockchain for supply chain security and data integrity

Blockchain ledgers, with their immutable records and cryptographic consensus, can assure stakeholders that no one has tampered with supply chain data—feed provenance, antibiotic usage logs, or animal movement records. Smart contracts can automate approvals and trigger alerts if anomalous data is detected. Still, blockchain adoption involves performance overhead, interoperability issues, and the need for collective agreement on standards. It is not a silver bullet but can complement other measures, especially for traceability-sensitive products like specialty cheeses or organic eggs.

### 6.3 Privacy and privacy-enhancing technologies (PETs) in livestock farming

As farms collaborate with off-site data analysts or share information with breeding cooperatives, PETs allow them to gain insights without exposing raw data. Techniques like homomorphic encryption or secure multiparty computation can compute statistical or AI model results without revealing sensitive inputs. By ensuring data privacy, PETs encourage data sharing that fuels innovation while preserving confidentiality and competitive advantages.

### 6.4 Secure device provisioning and hardware-level attestations

Securing the supply chain of IoT devices from manufacture to deployment ensures that no malicious components enter the farm environment. Hardware-level security features—like trusted execution environments (TEEs) and secure boot processes—prevent unauthorized firmware from running. Device attestation protocols enable verification that devices remain in a known-good state. These measures raise the bar for attackers, who must now subvert hardware protections rather than exploiting software alone.

### 6.5 Edge computing and fog security models

Shifting critical data processing to the farm (edge computing) reduces dependence on cloud services and bandwidth. Edge nodes can store offline backups, run IDS locally, and maintain minimal functionality during connectivity outages or cyber incidents. By distributing computation and storage, edge architectures ensure that a single compromised cloud server does not cripple entire operations. This approach also supports fail-safe modes where essential animal care functions continue even if external systems fail.

## 7 Integrating cybersecurity with biosecurity and food safety protocols

### 7.1 Harmonizing cyber and biosecurity measures

Livestock operations have longstanding biosecurity protocols to prevent disease entry and spread. Cyber threats can be conceptualized similarly—digital pathogens infiltrating networks and “infecting” systems. Creating integrated incident response plans that treat cyber intrusions as hazards akin to biological pathogens ensures rapid containment. Employees already trained in biohazard containment might more readily adopt similar mindsets for cyber containment.

### 7.2 Embedding cybersecurity in HACCP and other food safety frameworks

HACCP principles identify, monitor, and control hazards at critical points. By introducing digital hazards into HACCP analyses, farms ensure that key points (like sensor-based microbial detection steps) remain reliable and authentic. Regular audits test not just physical sanitation procedures but also data integrity checks. Incorporating cybersecurity into HACCP fosters a holistic risk management culture that treats digital anomalies as seriously as bacterial contamination.

### 7.3 Verification of data authenticity in precision livestock farming

Data authenticity verification methods—digital signatures, cross-referencing sensor readings, employing redundant sensors—can detect tampering. For instance, if feed consumption data from a silo conflicts with weight gain metrics consistently, it may signal malicious data injection. Ensuring authenticity instills confidence in predictive models and validates the trust-based relationships among farmers, technicians, and external experts.

## 8 Human-centric cybersecurity: training and capacity building

### 8.1 The human element in cyber risk

No amount of encryption or blockchain can prevent a breach if an employee unknowingly grants an attacker access. Humans remain the first and last line of defense. Farmers and workers must understand that cybersecurity failures can directly harm animals, yield, and finances. By internalizing cybersecurity as integral to animal care and business continuity, human actors become vigilant defenders rather than weak links.

### 8.2 Designing effective training modules for farm operators and staff

Training must be contextualized, repetitive, and interactive. Phishing simulations can teach staff to spot suspicious emails. Demonstrations that link security incidents to tangible animal welfare or financial losses underscore the urgency. Incorporating scenario-based learning and gamification can improve engagement, retention, and the willingness to report suspicious activities.

### 8.3 Upskilling and reskilling in rural environments

Rural communities may lack cybersecurity professionals or training centers. Extension services, agricultural universities, or industry bodies can fill this gap by offering on-site workshops, online courses, and certifications. Public-private partnerships could fund “cyber coaches” who assist multiple farms in a region, ensuring even smaller operations gain access to expertise and support.

### 8.4 Public awareness and transparency

Consumers, media, NGOs, and policymakers must understand that cybersecurity is not abstract but fundamental to ensuring safe, ethical, and reliable food. Transparent communication about preventive measures and incident responses can build public trust. Responding openly and swiftly to breaches, explaining lessons learned, and outlining corrective steps can prevent panic and speculation.

## 9 Policy, regulation, and standardization efforts

### 9.1 International, national, and regional regulatory landscape

Diverse regulatory landscapes hinder unified progress. The EU's GDPR prioritizes data protection but not necessarily operational data integrity. The U.S. FSMA focuses on food safety but lacks explicit cybersecurity mandates. Cross-border coordination, possibly through FAO or Codex Alimentarius frameworks, could spur agricultural cybersecurity standards. Achieving consensus is complex, as countries vary in digital readiness, priorities, and political will.

### 9.2 The role of industry associations and non-profits

Producer cooperatives, dairy boards, and poultry industry associations can develop tailored guidelines, sponsor educational programs, and advocate for policies that reflect on-the-ground challenges. Non-profits focused on rural development or animal welfare can urge inclusion of cybersecurity criteria in sustainability certifications or welfare audits, ensuring a holistic approach that aligns with social and ethical values.

### 9.3 Balancing innovation with compliance and liability

Overly strict mandates may stifle the entrepreneurial spirit driving agricultural innovation, while lax regulations risk catastrophic failures. A balanced approach might use performance-based standards, encourage voluntary adoption of recommended controls, and offer liability relief for farms that comply with recognized frameworks. Clarity on liability—if a breach occurs despite best efforts—builds confidence in investing in security.

### 9.4 Incentivizing cybersecurity investments through policy instruments

Governments can provide tax incentives for purchasing secure IoT equipment, subsidize farm-level cybersecurity audits, or fund pilot projects demonstrating advanced defenses. Insurers can factor cybersecurity compliance into premium calculations, rewarding proactive farms. Such incentive structures align economic rationales with security goals, accelerating sector-wide improvements.

## 10 Collaboration and stakeholder engagement in agricultural cybersecurity

The complex and evolving nature of cybersecurity threats in agriculture necessitates a collaborative approach involving diverse stakeholders. Public-private partnerships, international organizations, and cross-sector initiatives play crucial roles in developing and implementing robust cybersecurity measures for the agricultural sector.

Public-private partnerships (PPPs) have emerged as a vital mechanism for addressing cybersecurity challenges in agriculture. These partnerships leverage the strengths of both government agencies and private sector entities to develop comprehensive security solutions. For instance, the Food and Agriculture Information Sharing and Analysis Center (FA-ISAC) in the United States exemplifies a successful PPP model (FAO, 2017). The FA-ISAC facilitates the sharing of threat intelligence and best practices among food and agriculture sector stakeholders, enabling rapid response to emerging cyber threats. Similarly, the European Union Agency for Cybersecurity (ENISA) has established collaborative frameworks that bring together government bodies, agricultural technology providers, and farmers to enhance the sector's cyber resilience (ENISA, 2021).

International organizations play a pivotal role in promoting cybersecurity standards and fostering global cooperation in agricultural cybersecurity. The Food and Agriculture Organization (FAO) of the United Nations has recognized the importance of digital security in its efforts to modernize agriculture and ensure food security. The FAO's Digital Services Portfolio includes initiatives to strengthen cybersecurity awareness and capacity building in member countries. Similarly, the World Organization for Animal Health (OIE) has incorporated cybersecurity considerations into its guidelines for veterinary services, acknowledging the increasing reliance on digital systems in animal health management (Bissadu et al., 2024; EFSA Panel on Animal Health and Welfare, 2012).

Successful collaborative initiatives in agricultural cybersecurity demonstrate the power of multi-stakeholder engagement. The Precision Agriculture Connectivity Task Force, established by the U.S. Federal Communications Commission, brings together farmers, technology providers, and policymakers to address connectivity and security challenges in smart farming. This task force has been instrumental in identifying cybersecurity gaps and proposing solutions tailored to the unique needs of the agricultural sector. In Europe, the Internet of Food and Farm 2020 (IoF2020) project exemplifies a large-scale collaborative effort involving 123 partners from 22 countries. The project not only focuses on developing innovative IoT solutions for agriculture but also emphasizes the integration of robust security measures throughout the development process. Case studies of successful collaborations highlight the tangible benefits of stakeholder engagement in agricultural cybersecurity. For example, the Australian Cyber Security Centre's partnership with the grains industry led to the development of sector-specific cybersecurity guidelines, helping farmers protect their operations from digital threats. In the Netherlands, a collaborative initiative between Wageningen University & Research and several technology companies resulted in the creation of a secure data-sharing platform for precision agriculture, addressing farmers' concerns about data privacy and security.

Despite the clear benefits, fostering collaboration across diverse stakeholders in agricultural cybersecurity faces several challenges. One significant hurdle is the varying levels of cybersecurity awareness and technical expertise among stakeholders. While large agribusinesses may have dedicated IT security teams, small-scale farmers often lack the resources and knowledge to implement sophisticated cybersecurity measures (Albrechtsen and Hovden, 2010). Bridging this knowledge gap requires targeted education and training programs, as well as the development of user-friendly security solutions accessible to all farm sizes.

Another challenge lies in aligning the priorities and interests of different stakeholders. Government agencies may focus on national security and regulatory compliance, while private sector entities prioritize protecting intellectual property and maintaining competitive advantages. Farmers, on the other hand, are primarily concerned with operational continuity and protecting sensitive farm data (Eastwood et al., 2019). Balancing these diverse interests requires careful negotiation and the establishment of clear governance frameworks for collaborative initiatives.

Data sharing and trust issues also pose significant challenges to collaboration in agricultural cybersecurity. The sensitive nature of farm data, including production metrics, financial information, and proprietary techniques, makes some stakeholders hesitant to share information, even for security purposes (Bewong et al., 2023; Kayikci and Khoshgoftaar, 2024; Šestak and Copot, 2023). Overcoming these concerns requires the development of secure data-sharing protocols and the establishment of trust among participants in collaborative networks.

The global nature of agriculture and its supply chains introduces additional complexity to cybersecurity collaboration. Differences in regulatory environments, technological infrastructure, and cultural attitudes toward cybersecurity across countries can hinder international cooperation (Bingen and Busch, 2006). Harmonizing approaches and standards across borders is essential for creating a cohesive global framework for agricultural cybersecurity.

To address these challenges and enhance collaboration, several strategies can be employed. First, establishing clear communication channels and regular forums for dialogue among stakeholders can help build trust and facilitate the exchange of ideas and best practices. The creation of sector-specific information sharing platforms, similar to the FA-ISAC model, can provide a structured approach to collaboration (Skopik et al., 2016). Second, developing standardized frameworks and guidelines for agricultural cybersecurity can provide a common language and set of objectives for diverse stakeholders. Third, incentivizing participation in collaborative cybersecurity initiatives through policy measures, such as tax incentives or preferential access to government support programs, can encourage broader engagement, particularly from smaller agricultural operations. Fourth, investing in research and development focused on agricultural cybersecurity can drive innovation and create new opportunities for collaboration. Public funding for academic-industry partnerships in this field can stimulate the development of tailored security solutions for the agricultural sector.

Finally, promoting cybersecurity education and awareness programs specifically designed for the agricultural community can help bridge the knowledge gap and foster a culture of security across the sector. Extension services and agricultural education institutions can play a crucial role in disseminating cybersecurity knowledge to farmers and rural communities (Bada et al., 2019). By leveraging public-private partnerships, international cooperation, and multi-stakeholder initiatives, the agricultural sector can develop comprehensive and effective strategies to address evolving cyber threats. While challenges exist in fostering collaboration across diverse stakeholders, targeted efforts to build trust, align interests, and share knowledge can create a more resilient and secure digital agricultural landscape.

## 11 Gaps in the literature and research opportunities

The rapid digital transformation in dairy and poultry farming underscores several critical research gaps in cybersecurity, highlighting the need for focused investigations to guide effective and context-specific strategies. While significant progress has been made in identifying potential threats, many practical and theoretical issues remain insufficiently explored, presenting opportunities for impactful interdisciplinary research.

Foremost among these gaps is the limited availability of longitudinal studies that comprehensively evaluate cybersecurity measures' implementation and effectiveness over extended periods. Current research often provides isolated snapshots of practices without exploring long-term adaptation, sustainability, or resilience to evolving cyber threats. Future research should investigate how cybersecurity practices evolve within farming communities, assessing the long-term efficacy of continuous training programs, changes in staff behavior, and adaptations to emerging digital vulnerabilities. Such studies will yield insights critical for creating sustainable cybersecurity practices that align closely with operational realities.

Another significant limitation is the sparse understanding of the economic implications associated with cybersecurity measures. Current literature predominantly addresses technical solutions without thoroughly analyzing the broader financial consequences of cyber incidents, including both direct costs (such as ransom payments or immediate production losses) and indirect impacts, such as long-term reputational damage and market positioning. Future research should undertake comprehensive cost-benefit analyses examining how cybersecurity investments affect farm profitability, insurance premiums, and overall market competitiveness. Clarifying these economic dimensions can effectively motivate farmers toward proactive cybersecurity investments.

The intersection of cybersecurity with animal welfare represents another critical yet under-investigated area. While digital systems enable detailed monitoring of animal health, little research exists on the direct welfare consequences of cybersecurity breaches, such as compromised environmental controls or automated management systems. Investigations should explicitly assess how cybersecurity incidents impact livestock welfare, providing empirical evidence to integrate cybersecurity considerations into biosecurity and welfare frameworks. Additionally, exploring ethical dimensions around digital surveillance technologies—including privacy concerns and data usage—will be essential for responsibly navigating future advancements.

A further notable gap is the absence of standardized metrics for evaluating cybersecurity effectiveness within agricultural settings. Current practices lack clear benchmarks for comparing cybersecurity performance across different operations or regions. Developing specific Key Performance Indicators (KPIs), such as detection response times, incident mitigation costs, or improvements in stakeholder trust, can provide objective measures of cybersecurity effectiveness. Such standardized metrics would enable farms to benchmark performance, adopt best practices, and continuously refine their strategies based on concrete, measurable outcomes linked directly to animal welfare and operational effectiveness.

Finally, interdisciplinary research is urgently required to holistically address cybersecurity challenges in agriculture. Effective cybersecurity solutions require collaboration among cybersecurity specialists, animal scientists, sociologists, economists, and ethicists. By integrating perspectives on socio-economic factors influencing technology adoption, ethical dimensions of data usage, and animal welfare considerations, comprehensive and practical frameworks can be developed. Real-world pilot studies testing innovative technologies—such as quantum-resistant encryption, secure blockchain traceability, and edge-computing architectures—can provide empirical validation of interdisciplinary solutions, guiding best practices for securing digital livestock operations in an increasingly interconnected agricultural landscape.

## 12 Conclusions

The digital transformation of livestock farming in dairy and poultry sectors offers substantial opportunities while simultaneously posing significant cybersecurity challenges. With increased adoption of automated systems, IoT devices, and data-driven analytics, the potential for cyber threats escalates, expanding attack surfaces and introducing vulnerabilities specific to agricultural contexts. Addressing these complex threats demands tailored cybersecurity strategies, particularly for interconnected automated systems critical to dairy and poultry operations. Emerging innovations, such as edge computing-enabled smart agriculture, promise enhanced data security by reducing latency and mitigating cloud-associated vulnerabilities, thus improving real-time decision-making capabilities. Yet, advancing cybersecurity resilience requires targeted efforts to address prominent gaps, including the need for longitudinal studies evaluating cybersecurity effectiveness, comprehensive economic impact assessments, standardized metrics, and deeper integration of cybersecurity with existing biosecurity and food safety frameworks. Additionally, securing the AI and machine learning systems employed for predictive analytics and animal health management warrants dedicated interdisciplinary investigation. Strengthening cybersecurity across digital livestock systems holds profound societal benefits, such as bolstered food security, reinforced consumer confidence, enhanced animal welfare, and greater economic stability in rural communities. Achieving these outcomes necessitates continuous research, practical pilot implementations, and extensive stakeholder collaboration involving farmers, technology developers, policymakers, and researchers. Through concerted, interdisciplinary efforts, the dairy and poultry sectors can establish comprehensive cybersecurity measures, ensuring a secure, ethical, and resilient future for agriculture within an increasingly interconnected and digitized global food supply chain.
